# Major histocompatibility complex complement (MHC) Bf alleles show trans species evolution between man and chimpanzee

**DOI:** 10.1038/s41598-023-42016-1

**Published:** 2023-10-04

**Authors:** Antonio Arnaiz-Villena, Ignacio Juarez, Alejandro Sánchez-Orta, José Manuel Martín-Villa, Fabio Suarez-Trujillo

**Affiliations:** 1 Departament of Immunology, School of Medicine, University Complutense of Madrid, Madrid, Spain; 2 Instituto de Investigación Sanitaria Gregorio Marañón, Madrid, Spain; 3 Departamento de Inmunología, Facultad de Medicina, Universidad Complutense, Avda. Complutense S/N, 28040 Madrid, Spain

**Keywords:** Diseases, Rheumatic diseases

## Abstract

HLA and disease studies by using single allele statistics have been fruitless during the last 40 years for explaining association pathogenesis of the associated diseases.Other approaches are necessary to untangle this puzzle. We aim to revisit complement alleleism in humans and primates for both studying MHC and disease association to complotypes and extended MHC haplotypes in order to also explain the positive directional selection of maintaining immune response genes (complement, MHC adaptive and MHC non-specific genes) that keeps these three type of genes together in a short chromosome stretch (MHC) for million years. These genes may be linked to conjointly avoid microbes attack and autoimmunity. In the present paper, it is obtained a new Bf chimpanzee allele, provisionaly named Patr-Bf*A:01,that differs from other Bf alleles by having CTG at eleventh codon of exon 2 in order to start the newly suggested methodology and explain functional and evolutionary MHC obscure aspects. Exons 1 to 6 of Ba fragment of Bf gene were obtained from chimpanzee. This new chimpanzee Factor B allele (Patr-Bf*A:01) is to be identical to a infrequent human Bf allele (SNP rs641153); it stresses the strong evolutive pressure upon certain alleles that are trans specific. It also may apply to MHC extended haplotipes which may conjointly act to start an adequate immune response. It is the first time that a complement MHC class III allele is described to undergo trans species evolution,in contrast to class I and class II alleles which had already been reported . Allelism of complement factors are again proposed for studying MHC complement genes, complotypes, and extended MHC haplotypes which may be more informative that single MHC marker studies.

## Introduction

The complement system alternative pathway is the most ancient of the three complement pathways (alternative, Ig-mediated and lectins pathways) from an evolutionary point of view^1^. C3 Convertase on alternative pathway is composed of C3b and Factor B (Bb fragment) proteins^2^. Several diseases have been linked to some allelic Factor B (Bf) polymorphism extant in Ba fragment^3–7^. Once C3b bounds to cell microbial or other surface, it can bind Bf protein, which is cleaved by Factor D to Ba and Bb fragments^1^, establishing BbC3b convertase on human pathogens surface. This convertase is labile but properdin (Factor P) serum protein stabilizes it. Ba has in fact an unknown function even if its alleles have been linked to diseases. C2 and Bf are close together since they were duplicated: Bf gave rise to C2 on the Major Histocompatibility Complex region of humans and apes^8^ and are classified as MHC class III genes. MHC segments and haplotypes may be highly conserved and usually their genes are conjointly trasmited^8^. Recently, we have stressed the striking evolutionary pathway of three type of genes being together in MHC for at least 40 million years in the ape line: adaptive immune response genes (i.e.: MHC class I and II), natural immune genes (i.e.: C2, C4 and Bf), and modulatory non-classical class I genes (MHC-G, -E and -F). This could be attributed to that a conjoint function of the extended MHC haplotypes genes there exists. The study of MHC haplotypes could be more appropriate to uncover MHC-linked diseases pathogenesis than the routine of using MHC locus single alleles. A wider study on C2, C4 and Bf alleles in primates may give a clue to guide the yet very long search to understand the association of MHC and disease pathogenesis^8^.

It is now recognized that single classical HLA genes and disease association has been studied since 1967^9^and no firm pathogenethic mechanisms have been yet established; this is also true for non classical HLA genes (i.e.:HLA-G)^10,11^.On the other hand, C2,C4 and Bf complement alleles ceased to be studied thoroughly by researchers and we propose that HLA haplotypes (several genes together) and also complotypes should again be studied in view of the fruitless results of statistical HLA single gene studies and disease association^10–13^.

In the present paper,we uncover a new Factor B allele DNA sequence since intensive  complement genes alleles studies may be useful for the study of both HLA and diseases association, including C4^14^ and we now start revisiting this abandoned research area.We present a new Bf allele in chimpanzee (*Pan troglodytes*) and provisionally name it as Patr-Bf*A:01.

## Material and methods

This study was performed with EBV-immortalized B-cell cell lines from chimpanzees kindly provided by Peter Parham. The amplification of the Ba fragment of the Factor B gene was carried out once the RNA was extracted from the cell cultures and cDNA synthetized from it and subsequently sequenced. The amplification was carried out with the specific primers Bf Hu 5′: 5′-AACTCTGCCTGATGCCCTTTATC-3′, and Ba Hu 3′: 5′-ATGGAGCCTGAAGGGTCCAGGA-3’. The PCR products were ligated into a cloning vector (pMOSblue Kit) (Amersham, Buckinhamshire, England, UK) with which competent bacteria were transformed. Once the recombinant clones were purified, they were sequenced by Sanger sequencing procedure.

### Ethical approval

This study was conducted according to the Declaration of Helsinki by the Ethics and Research Committee of University Complutense.

## Results

**Figure 1:**
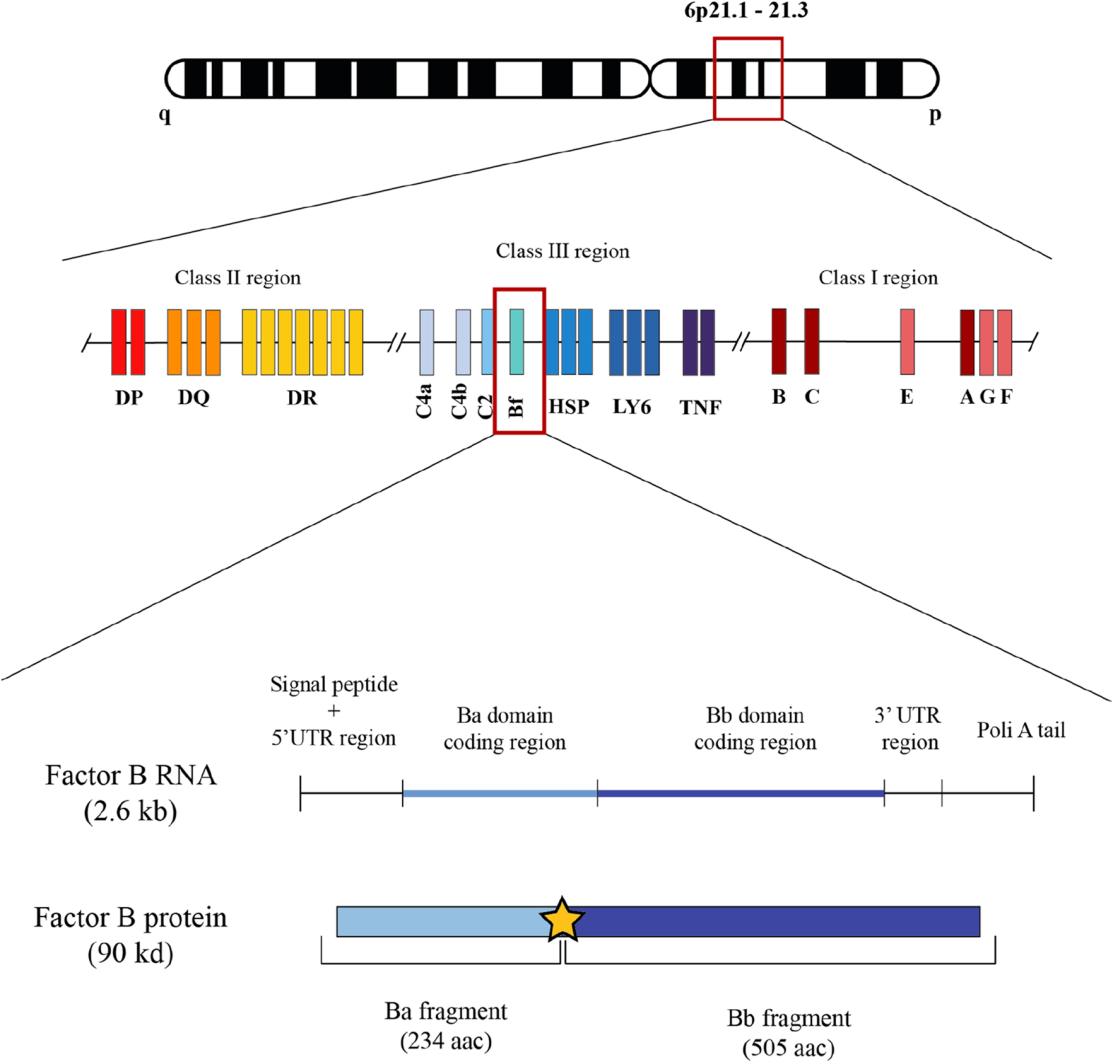
HLA genes are located in the short arm of human chromosome 6. Complement genes (C4a, C4b, C2 and Bf) are located in the class III HLA region, between class I region (telomeric) and class II region (centromeric). Factor B RNA is translated into a 90 kd protein which is cleaved by Factor D in the catalytic site (yellow star) giving rise to separated Ba and Bb functional fragments. Primates^14^, mouse^15^and birds ^16,17^also bear complement within their MHC complex.

**Figure 2:**
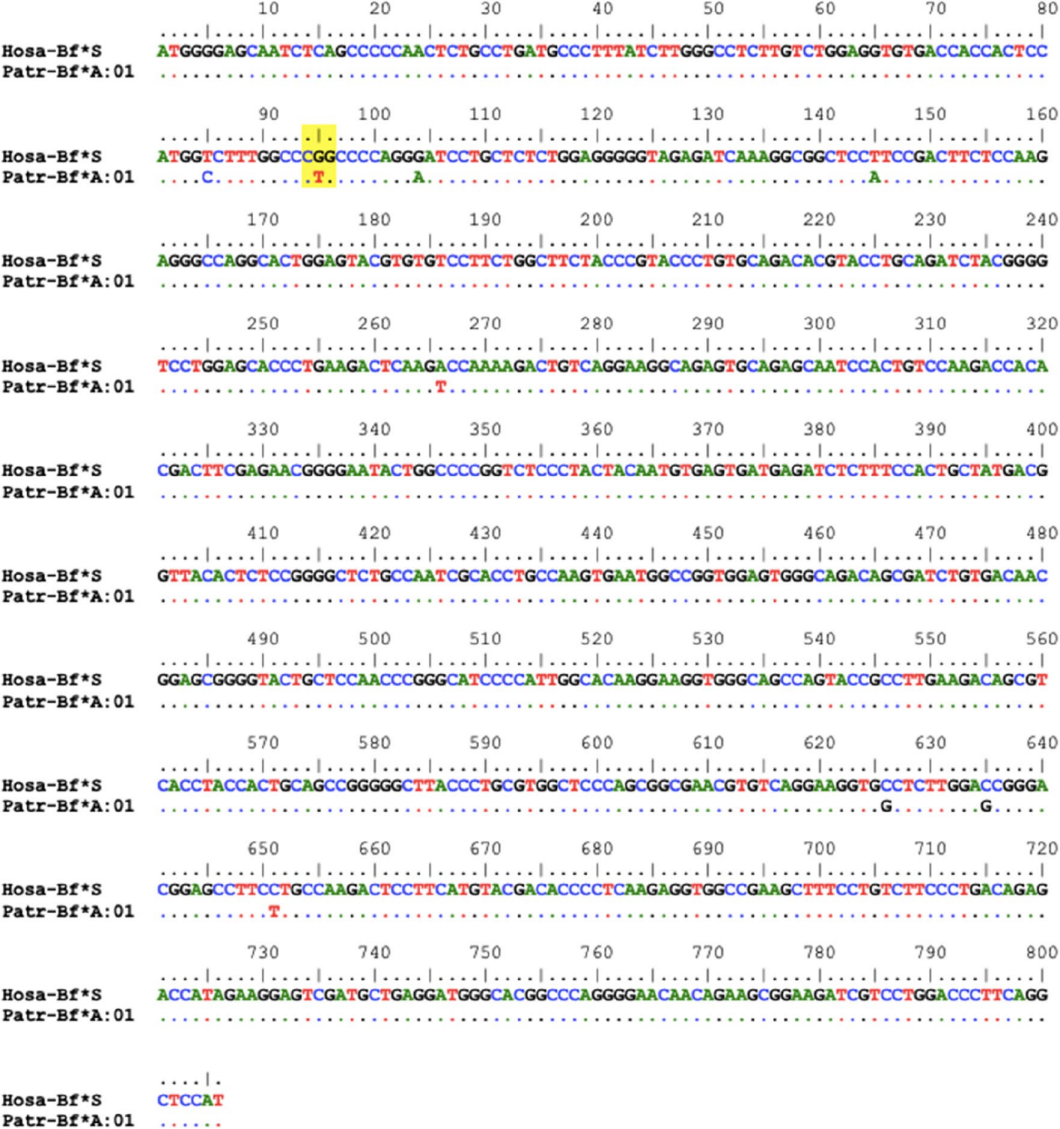
Sequence of the provisionally designed Patr-Bf*A:01 allele aligned with human Hosa-Bf*S^18^. Eleventh codon of exon 2 (position 7 of the mature protein, bases 94–96 of the complete coding sequence) is remarked in yellow: while in S alleles it is CGG, in the new allele it is CTG.This chimpanzee Bf allele is identical to that of human Bf rs641153 ,variant CGG to GTG (R32L).This DNA sequence is deposited in GenBank with accession number AF013267.

The obtained sequences were analyzed with the MEGA 7 and BioEdit programs; only exons (1–6) were taken for the study.For analysis, the sequence was aligned with the human Hosa-Bf*S sequence (Fig. 2). For the nomenclature of the new allele, the standard procedure has been followed: the first four letters (Patr) correspond to the names of the genus and species (*Pan troglodytes*), followed by a separator (-) and the gene (Bf*). It has been shown that the only difference at the DNA level found between the S, FA and FB subtypes of the Bf gene consists of a change in the eleventh codon of exon 2 (position 7 in the mature protein, bases 94 to 96 of the complete coding sequence): CGG in the case of Bf*S, CAG in Bf*FA, and TGG in Bf*FB^18^. In the present new allele described, the codon at position 11 of exon 2 is CTG (Fig. 1), which does not correspond to any of those mentioned above: thus a new allele is found. For this reason, we have provisionally assigned the letter "A" followed by the number “01” to indicate that this is the first allele to be described at the DNA level with this type of variation in exon 2 coding sequence (see Fig. 2 footnote).

The DNA sequence of this allele is deposited in the GenBank database with accession number AF013267 and also shown in Fig. 2.

On the other hand, the rs641153 polymorphism in man Bf codon 32 was described : CGG→CAG (R32Q) variant. CGG in codon 32 which codifies for arginine and identifies the Bf*S alleles and CAG codifies for a glutamine and identifies the Bf*F alleles (https://www.ncbi.nlm.nih.gov/snp/rs641153#variant_details) (https://www.ensembl.org/Homo_sapiens/Variation/Phenotype?db=core;g=ENSG00000243649;r=6:31945650-31952086;v=rs641153;vdb=variation;vf=167620393)^18,19^. A third human Bf variant was later described CGG→CTG (R32L) in one Caucasian individual only; it is a infrequent human Bf variant which has identical DNA polymorphic sequence to the chimpanzee allele described in this paper (https://www.ncbi.nlm.nih.gov/projects/SNP/snp_ss.cgi?subsnp_id=ss244317409) (https://gnomad.broadinstitute.org/variant/6-31914180-G-T?dataset=gnomad_r2_1).

This is the first described case of allelic trans species evolution in human/ primates MHC complement class III genes. MHC class II trans specific alleles between human and primates have been described^20^ and also between MHC class I different bird species^21^.

## Discussion

We have insisted in our recent works^10,11^ that single MHC alleles and disease studies based in statistical associations have not uncovered classical HLA alleles linked to disease pathogenesis (i.e.: the fruitless last 50 years of HLA and disease associations, since Amiel studies in 1967). Thus, we have proposed to go back and study complotypes^22^ and extended HLA haplotypes^12^, and also MHC evolution in a primates model^10,11^, which may give us a clue of why complement, classical and non-classical HLA class I genes and other non adaptive immune response genes have evolved together in the same chromosome stretch of each species. Also, it could save time at studying endless and fruitless MHC (HLA) non-classical class I associations with diseases^11^.

### Factor B alleles linked to diseases

This hunan rs641153 human complement Bf is linked to eye macular degeneration (SNPedia rs641153). Juvenile Rheumathoid Arthritis has been found to be significantly linked to Bf*F1 regarding to a certain clinical form^23^, whether or not this is due to the linkage disequilibrium with HLA-DRB1*03:01 is uncertain. Bf*F1 is associated with some autoimmune diseases like type 1 diabetes ^3^ and systemic lupus erythematosus^4,5^. A role for the alternative complement pathway in Systemic Lupus Erythematosus (SLE) has been shown^24^. Bb fragment is postulated to be important in SLE severity detection; the Bb fragment was not detected in the activated plasma samples from SLE patients, which suggest that activation of the alternative complement pathway may be a marker for severe SLE and that the Bb fragment may be playing a role in the development of this severe pathologic condition^21^.

Bf*F1 is also important in severe SLE^6^, whether these associations are primary or not is not clear, but HLA-DRB1*03:01 was quickly blamed, which is in linkage disequilibrium with Bf*F1^4,5,7^.C4 alleles may also be useful to study autoimmunity association^22,23^. In summary, Bf*F1 could be associated to autoimmune disease either by itself or together with HLA extended haplotypes containing HLA-DRB1*03:01 and Bf*F1.

### Complement genes and trans species Bf allele evolution

Birds bear complement, MHC and other immune genes (the MHC-B in chicken micro-chromosome 16)^16,17^, like also demonstrated in Old World primates lineages in their respective chromosome sites. This 40 million years of a set of genes conservation for specific and non-specific immunity is explained by the existence of a positive directional selective evolution by unknown reasons. However, it suggests that this set of genes (and alleles) work together to maintain individual ‘self’ against microbes or against immune system itself (autoimmunity), and it is not a fact that has occurred by chance^11^. Disruption of the proper set of genes (alleles) may lead to an individual life threat either by infection and/or autoimmunity^10,11^. This is further supported by the chimpanzee/human Bf allele trans species evolution described in this paper: it is the first time that a complement class III MHC allele is observed to be trans specific showing that strong evolutive pressures may be acting upon the allele and its extended MHC haplotipe. These are often inherited in block together with neighbouring genes (and alleles) stressing that MHC haplotypes with different genes and alleles may be important for survival and immune defense.

These observations are because we propose to study sets of MHC genes (and alleles) evolution and/or complotypes in order to try to counteract/mend the deceiving single MHC allele study^10,11^ in addition to the lack of explained MHC linkage to disease physiopathologies that could explain this linkage after more than 50 years study on HLA and disease.

## Data Availability

The DNA dataset generated/analyzed during the current study is shown in Fig. 2. It is als deposited in GenBank with accession number AF013267 .
